# Automated 3D measurements of the aortic length using the Hough transform

**DOI:** 10.1186/1532-429X-13-S1-P46

**Published:** 2011-02-02

**Authors:** Alain de Cesare, Muriel Lefort, Claire Pellot-Barakat, Elie Mousseaux, Alain Herment, Frederique Frouin

**Affiliations:** 1 Inserm/UPMC, Paris, France; 2 AP-HP, Paris, France

## Introduction

A 3D estimation of the length of the aortic arch using sagittal and axial acquisitions is proposed and compared with the conventional 2D method using a single view of the aorta.

## Purpose

The aortic length is required to accurately estimate the Pulse Wave Velocity (PWV), which is an important index of aortic stiffness. Moreover, both the aortic length and the PWV are modified with age. The 3D approach was motivated to better take into account the geometry of the arch.

## Methods

The conventional 2D method is based on a single view of the aorta which is combined with phase contrast acquisitions to define the starting and stopping points of the arch, where velocities are measured. For the 3D measurement, SSFP axial and sagittal slices including the aortic arch are considered. Two operators measured the 3D length of the arch by delimiting markers on the different views. The visual control was supplemented by a 3D representation of the arch. Independently, the Hough transform adapted to circular shapes was used to define the lumen centers on the same views and to thus automatically define markers in the aortic arch.

## Results

**Figure 1:**
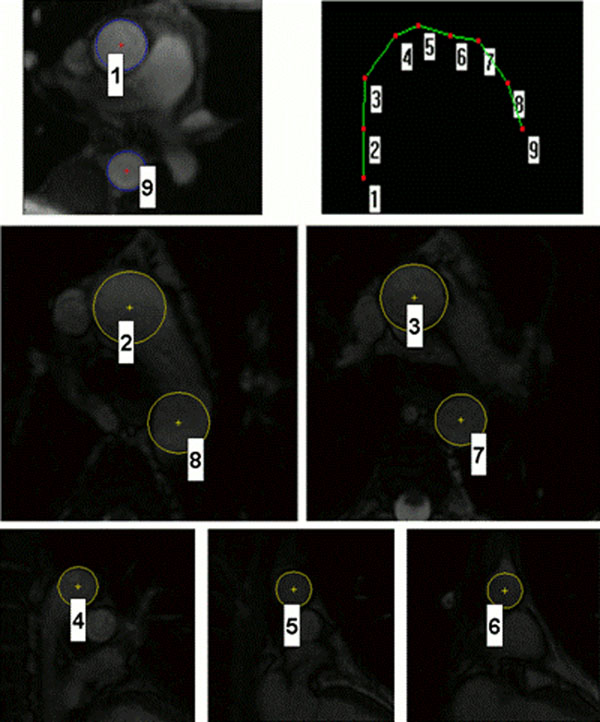
Automated definition of markers for phase contrast images, axial slices, and sagittal slices, and corresponding 3D visualization.

Studies from 20 volunteers (22 to 67 years old) were processed manually and by the automated approach, providing three 3D measurements of the aortic arch. The 2D measurements were always statistically inferior to the 3D ones. Three dimensional series of measurements were well correlated (r >0.93), with a slope close to 1. For manually defined markers, the inter operator variability was 4%; while the variability between the automated and manual methods were 3.8% and 4.4% for the first and second operators, respectively. The mean bias between the two operators was about 4 mm, while it was about 2 mm between each operator and the automated method. Finally, using the three methods, the 3D lengths of the aortic arch were well correlated with age (r>0.63). Figure 1.

## Conclusions

The automated procedure is fast, efficient, and robust. Its incorporation into the ART-FUN software platform could help in a larger automation of functional and morphological analysis of the aortic arch. To further improve the estimation of the aortic length, registration strategies are under investigation to compensate for possible misalignments between the successive apneas that are required for the different acquisitions.

